# Design and Implementation of a Wireless Sensor Network-Based Remote Water-Level Monitoring System

**DOI:** 10.3390/s110201706

**Published:** 2011-01-28

**Authors:** Xiuhong Li, Xiao Cheng, Peng Gong, Ke Yan

**Affiliations:** 1 College of Global Change and Earth System Science, Beijing Normal University, Xinjiekouwai Street No.19, Beijing 100875, China; 2 State Key Laboratory of Remote Sensing Science, Jointly Sponsored by the Institute of Remote Sensing Applications of Chinese Academy of Sciences and Beijing Normal University, P.O. Box 9718, Beijing 100101, China; E-Mails: gong@irsa.ac.cn (P.G.); irsa2008@sina.com (K.Y.)

**Keywords:** water-level monitoring system, wireless sensor, remote monitoring system, Poyanghu Lake

## Abstract

The proposed remote water-level monitoring system (RWMS) consists of a field sensor module, a base station module, adata center module and aWEB releasing module. It has advantages in real time and synchronized remote control, expandability, and anti-jamming capabilities. The RWMS can realize real-time remote monitoring, providing early warning of events and protection of the safety of monitoring personnel under certain dangerous circumstances. This system has been successfully applied in Poyanghu Lake. The cost of the whole system is approximately 1,500 yuan (RMB).

## 1. Introduction

Water-level monitoring has been widely used to reduce the danger of flooding disasters, ensuring the safety of ship channels and monitoring the aquatic environment. Field monitoring by people is still used, but sensor monitoring is being adopted by more monitoring stations. Currently some commercial product-based monitoring systems can be found in China, like the JWR-SJ water-level monitor from the Beijing Jiawer Co. [1] or the HG35-QSW water-level monitoring system of the Beijing Zhongxi Co. [2]. Others are water-level monitoring systems based on wireless sensor networks such as the DATA86 water-level monitoring system of Tangshanpingsheng Co. [3]. Similar monitoring products are also found overseas, such as TD-DIVER, an underground monitor by Holland Foreign Talent and YSI Level Scout, a water level monitor with large data storage capacity from Doppler U.S. However, these on-the-spot automated devices still need people to download the data in the field later, therefore, researchers have sought to design and implement remote water environmental monitoring systems. Some are based on wireless sensor networks through the transmission of sound waves [4]. Others are based on an *ad hoc* network of wireless nodes [5–7], or adopt handheld data gathering and return data to the server using a GPRS network. Due to the interference from the transmission distances among the wireless nodes of the sensor network, such monitoring systems are more applicable to small-scale water monitoring scenarios. Considering the vast territory and complicated hydrological environments of China, the applicability of the above methods is greatly limited.

Let us consider, for instance, Poyanghu Lake, located in the Hengshan shoals of China, where seasonal flooding occurs. A practical method is also needed to simultaneously monitor multiple ground water wells which distribute water. As a third example, many quake lakes were formed during the Wenchuan Earthquake in Sichuan Province of China on May 12th, 2008. The water-level of those quake lakes increased rapidly and threatened life and property, so there was an urgent need to monitor the water-levels quickly and accurately, but the appropriate Water Department still used field monitoring by people, which is very inefficient. In addition, the safety of the monitoring personnel was threatened by the aftershocks, landslides, and rainfalls, so it was hard to accomplish the water-level measurements. Under such circumstances, a remote water-level monitoring system could provide synchronized data directly to the concerned Water Department and implement real-time monitoring and provide early warning of the water-levels of quake lakes.

The remote wireless water-level monitoring system adopts the self-developed embedded ZKOS operating system in order to ensure an expansible and real-time system and also reduce the cost. The system timer is revised or adjusted according to the satellites synchronized through GPS chips, which will guarantee the synchronicity among the systems located in different regions. Considering most of the sites are in the field, a program is designed and developed so that the system can be updated remotely. The related software is also developed in the remote server to monitor and control the frequency of the system. Therefore, this system can be customized according to users’ inquiries. Currently, this system is being used to monitor the water-levels of Poyanghu Lake. It is also planned to use it to continuously monitor the degree of mineralization of groundwater and water-levels in Quzhou, Hebei Province, China.

## 2. System Design

### 2.1. System Introduction

**Figure 1.**
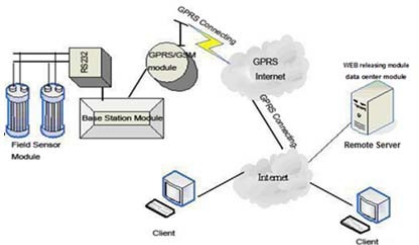
System structural framework.

The RWMS consists of the field sensor module, the base station module, the data center module and the WEB releasing module (Figure 1). The field sensor module acquires the real-time data of the water-level and sends the converted data to the MCU (Micro Control Unit). The base station module controls the field sensor module and transfers the data to the data center module by GPRS/GSM.

The data center module receives and stores data from the GPRS/GSM module and then transfers the data to the WEB releasing module where the data will be published.

### 2.2. Field Sensor Module

The field sensor module, composed of the water-level sensor and the electrical level exchange chip, is mainly used to obtain the field water-level data. The module collects the water-level information as an analog signal, converts it to a digital signal by a 12 bits A/D of high accuracy, and then sends it to the MCU.

### 2.3. Base Station Module

The base station module is the core of the RWMS. Comprised of the hardware and the software, it integrates the RTU (Remote Terminal Unit), MCU, and GPRS/GSM functions of the unit.

#### 2.3.1. The Base Station Module Hardware

**Figure 2.**
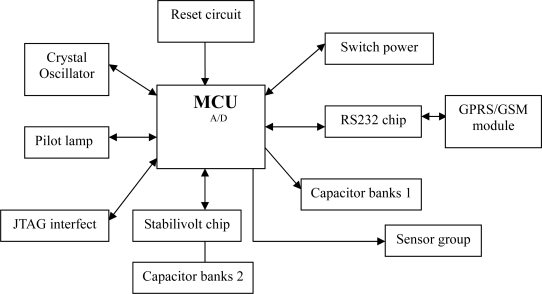
Hardware components of the base station module.

The base station module includes the MCU, reset circuit, capacitor banks, and serial expansion chip (Figure 2). MCU is the control core of the RWMS based on the wireless sensor networks. The embedded operating system, ZKOS (Zhongke Operating System), controls and implements the functions such as acquiring data, processing data, transferring data and so on. The ZKOS will be described in details in a later section. In the reset circuit, resistance and capacitance are in series and the reset circuit connects the RESET connections of the MCU. The capacitor banks can reduce the disturbances, such as high frequency, fluctuations, unsteady state and the power surges to ensure the MCU works normally. The base station module can provide five serial ports, eight A/D of 12 bits, two CAN buses and 10 common programmed I/O interfaces in order to conveniently connect to multiple different interface sensors. The acquired data can connect the GPRS/GSM module through the serial port and can be exchanged with data on the remote server through the GPRS/GSM module. The core hardware of the GPRS/GSM module adopts the integrated package product from the New Thinking Company. The software is highly reliable and self-developed based on the embedded ZKOS operating system.

#### 2.3.2. The Base Station Module Software

##### (1) Embedded operation system—ZKOS

**Figure 3.**
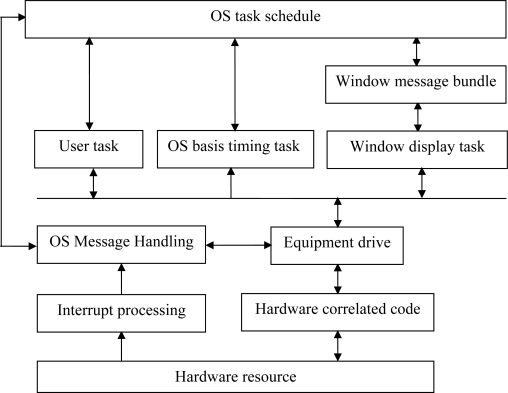
The software structure of ZKOS.

The embedded operation system is applicable to different single chips and features small code, less dependence on stacks, registers, timers and interrupters and being applicable to different single chips. The software structure of the embedded operation system ZKOS is shown in Figure 3.

##### (2) The ZKOS-based remote wireless monitoring system

All tasks performed by the embedded operation system ZKOS in the remote wireless water-level monitoring system can be classified into five types:

Task 1: All signals from the analog channel would be converted in a timely fashion into digital signals by A/D. The digital signals then would be converted into the true value. The timing must be above 10 μs and may be revised.
Task 2: The sample data of the water-level sensors can be acquired in time after the communication through all the data channels. The Timing must be above 0.1 ms and may be revised.
Task 3: The acquired data acquired should be packed up and transmitted to the remote Internet server in time by the GPRS module. The Timing must be above 1 min and may be revised.
Task 4: The working modes of the system or the corresponding operations can be set by the instructions derived from the text messages received by the GSM.
Task 5: To upgrade the program remotely.

##### (3) The remote upgrade task

The software of the system needs to be improved in order to solve some specific problems noted during the running. The current method is to download the procedures or to change the FLASH accessories on the spot. However, it may be hard to access the spots once the system has been set up in the field, therefore, it is a good method to remotely upgrade the program remotely using the GPRS network [8]. The online upgrade can be carried out by dividing the memory space of the FLASH in the MCU of the base station module and defining the data format transmitted by the upgrade file using IAP (Internet Access Provider).

The GPRS remote upgrade system consists of the server and the remote terminal. The server checks if there are new programs to upgrade. The remote terminal includes MCU and GPRS modules, which communicate through USART interfaces. The server and the remote terminal communicate through GPRS. When the software needs to be upgraded at the server, the upgrade bit will be marked and then the upgrade label and upgrade package will be sent back to the terminal via GPRS.

In the remote terminal there are two types of FLASH storage media: internal FLASH and external NAND-FLASH. The internal storage media is divided by IAP programs area and application programs area. The IAP programs are only executed to upgrade the software when the system starts and upgrade programs are needed. The application programs are executed to implement all the functions of the system when the system operates normally.

**Figure 4.**
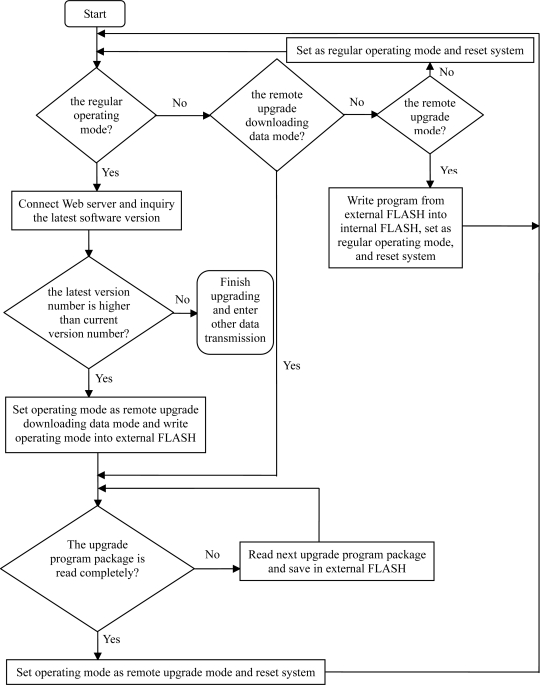
Flow chart of the remote upgrade task.

The external NAND-FLASH storage media can also be divided into two portions: program package areas and data storage area. The system will receive the program package from the GPRS module and save it into the program package areas. When receiving of the correct program package is confirmed, the system will be reset. The program will be written into the internal program package areas by IAP program. The system will operate normally after the second resetting. The flow chart of the remote upgrade process at the terminal is shown in Figure 4.

The system checks the upgrade label each time after being reset. If the upgrade bit is marked, then the system is operated by the IAP program. The program package of the external NAND-FLASH will be written into the internal FLASH program areas, then reset the system. If not marked, then the system is operated by the application program.

When the system is operated normally, if the upgrade label from the server is received, then the upgrade validation request is sent back to the server with the necessary information for upgrading, such as the number of program packages, the size, and the version number, *etc*. (such information can also be sent from the server directly to the terminal). Once the upgrade validation is confirmed, the upgrade program package starts to be received and written into NAND-FLASH. When receipt of the upgrade program package is finished and the validation code is correct, the system needs to be reset for the upgrade to occur.

### 2.4. Data Center

**Figure 5.**
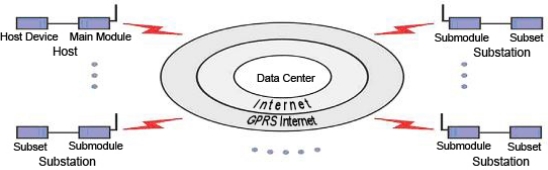
The communication of the GPRS/GSM module.

The data center is a software that manages the access of the GPRS/GSM module and the returned data. The data center communicates with the GPRS wireless network through the Internet under the TCP/IP protocol. Background automatic management and extensible analytic method are used in each GPRS/GSM module. This software has a simple operation interface and is highly adaptable to networks. The GPRS/GSM communication module is shown in Figure 5.

#### 2.4.1. Working Flow

**Figure 6.**
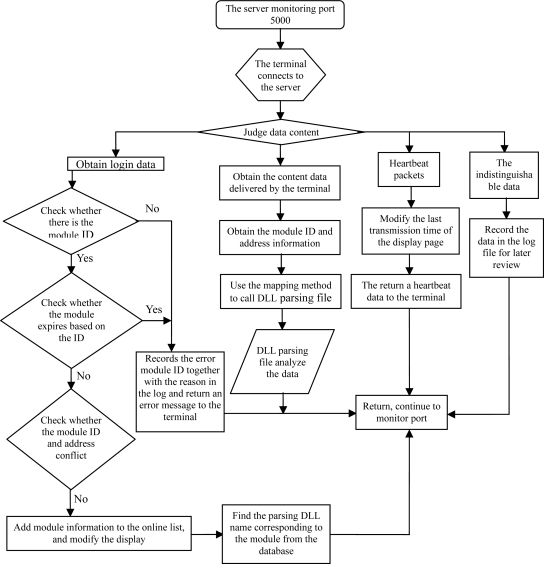
The working flow of the data center.

C-S structure is adopted for the server data center, as shown in the flow chart in Figure 6. The center also makes use of TCP-IP socket protocol in a communication protocol to achieve multi-block simultaneous communication in a multi-threaded manner.

The C-S (Client-Server) structure of the workflow is as follows:

C: a single module's connections links to the server through a fixed IP external network and a specified port number.
S: each terminal is assigned with a separate thread to receive and send data at terminals from the server, which is always in the monitoring state.
C: the module sends login information.
S: the server tests login information.
C: the module sends data
S: the server interprets the data through the specified analytical method call (class file) in accordance with the logging information.

#### 2.4.2. Introduction to Functions

(1). Online List: The online list shows the detailed information of the module, such as the IP address, the login time and the data update time after logging in the data center. The state of the GPRS/GSM module would be examined and the online list could be cleaned by the right-click shortcuts.

(2). Framework: The framework, a key of the data center, manages the module access, the categories of modules and the data reception. Each project is a unit to which one module belongs. To acquire the project data requires the addition of the corresponding DLL dynamic data analysis. The compiled DLL file could be put into the catalog of the executing program in the server. The GPRS/GSM module may modify the service life and the access permission to make the module more conveniently to be used by the project.

(3). Data Management: The module data of each project can be viewed. The data management interface is extensible, which makes it easier and simpler to display data.

(4). Setting the Data Center: The startup options of the data center include the default auto-running mode and the manual configuration mode. These two options make the startup more convenient. Furthermore, they both can meet different network environments, because the data center will be operated according to the user’s options once the server restarts.

#### 2.4.3. Connecting with the Data Publishing Module

**Figure 7.**
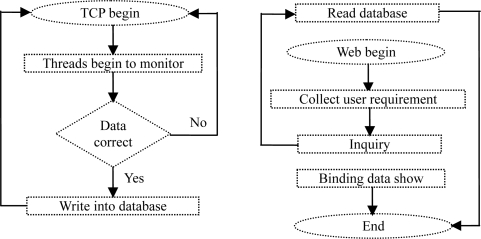
Flow chart of the data releasing module.

Multithreading monitoring of a certain terminal is carried out when the TCP background program of the data center starts. Once the data is transferred from the module to server, a single-threading will be set up to process the data and finally store the processed data in the database. In the data releasing module, when the user opens the Web page and searches the needed information, the program will collect the conditions input by the user, on basis of which the program inquires the data in the database. All the data up to the requirement will be bound to the display control (in form of data table or graph) and exhibited to the user. The program flow chart is shown in Figure 7.

### 2.5. Data Releasing Module

The data releasing methods for the module include the text message releasing mode and the WEB releasing mode.

#### 2.5.1. Text Message Releasing

The water-level data can be obtained by text message and the modality of obtaining data can be controlled in the RWMS. It is very practical where the Internet is inaccessible. The real-time data can be obtained and the modality of obtained data can be modified conveniently by the user by sending a pre-set text message to the mobile phone number bound to the GPRS/GSM module. Users can also receive data, set the time interval of data transfer and cancel the mobile phone number bound and so on by sending messages. Any spam messages can be blocked in the ZKOS by defining the password with a short message.

#### 2.5.2. WEB Releasing

The real-time water-level data can be acquired through Internet on the WEB releasing module. In view of the application and analysis of the historical data, three web releasing methods are designed, including map releasing, database releasing and the water-level variation trend chart.

1. Map Releasing: The Web electronic map is introduced in the RWMS to show the detailed locations, descriptions, and the maximum/minimum of water-level by adding the labels in the map. The map of China will be the default map, but the map can be customized through revising the scale and the sites in the background configuration file.
2. Database Releasing: The data obtained from RWMS is saved as specified data format in the designated database. The real-time database inquiry can be achieved through the database development, such as the site inquiry, the period inquiry and the matching inquiry.
3. Water-level Variation Trend Chart: The water-level variation trend chart of the designated site can be drawn in RWMS through choosing the site and the time period. The user can follow the water-level trend by appointing the last time as the collection time of the last piece of data.

### 2.6. The Custom-Made RWMS in Application

**Figure 8.**
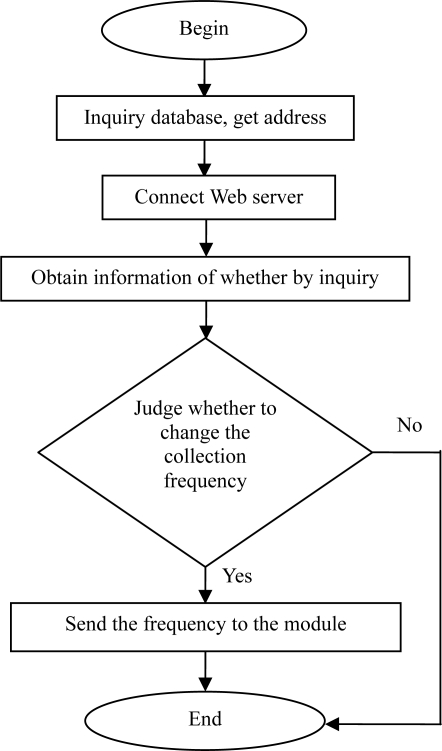
The flow chart of the underground water monitoring.

In RWMS, the functions of the software and the hardware can be expanded to meet the user’s requirements. In the plans to continuously monitor the groundwater levels in Quzhou, Hebei Province, China the monitoring frequency is combined with weather forecasting in the system design. The monitoring frequency of the system will increase automatically in case of heavy rainfall. Such work is undertaken by the data center and the data releasing module, as shown in the flow chart of the underground water monitoring (Figure 8).

### 2.7. Communication Description

A 2.4 G wireless Tx/Rx module for *ad hoc* network or the GPRS/GSM module of China Mobile’s GPRS network can be used in the wireless communication of the base station module. The latter has been adopted this time. The independent research and design module not only reduces the cost but also decreases the communication expenses greatly in a user-defined communication data format. The background server program makes the system stronger and better fit the specialized applications.

## 3. System Realization

**Figure 9.**
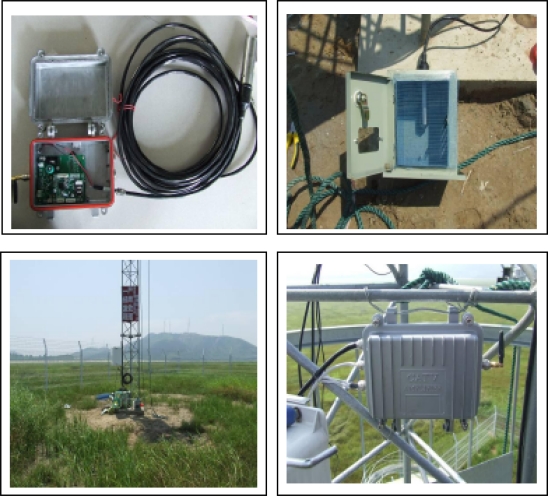
The installation pictures of the RWMS at Poyanghu Lake.

The RWMS of Poyanghu Lake is located in the shoals of Hengshan, Jiangxi Province, China. The coordinates of the site are 29°16′6.9″ N, 116°07′13.0″ E. Pictures of the installation are shown in Figure 9. The Jishan Shoals are encircled by the Dajishan Hills, Xiaojishan Hills and the Middle Dam. The shoals. measuring 3.5 km from south to north and 2.5 km from east to west, are covered by higherr vegetation such as sedge and mugwort and periodically flooded annually between May and September, thus forming a typical marsh ecosystem.

**Figure 10.**
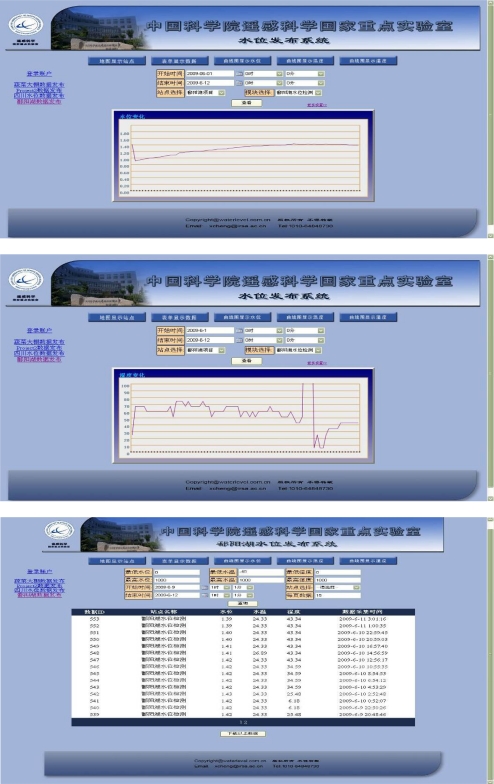
The Poyanghu Lake water-level releasing system.

The sampling interval of RWMS has been set into one hour in order to acquire the water-level data of the Poyanghu Lake speedily. The water-level releasing system in the Poyanghu Lake (Figure 10) can realize the map releasing, the database releasing and the water-level variation trend chart releasing.

## 4. Results

### 4.1. Time Delay

The time delay of RWMS includes the network delay, the data collection delay and the average data processing delay. The GPRS network delay in TCP/IP transmission mode changes from dozens of ms to hundreds of ms. The data collection may be delayed if the clock frequency of the data collection is above 4 ms. The RWMS uses the average value of the sampling data below 500 ms in order that the measured value may not be affected by any unexpected outside interference, such as wind, barometric pressure, and water, which may result in the changes of underwater pressure. The maximum delay of the system is around 1 second.

### 4.2. Precision

The RWMS collects the data by 12 bits A/D and adopts an oversampling method, which improves the accuracy of the system by increasing the digit of ADC to 14 through programs [9]. When the range of the sensor is L (0∼15 m in this system), the range of the output current is A_1_–A_2_ (4∼20 mA in this system). Assumed the ADC digit is n, then the highest resolution of sampling is L/2^n^* (A_1_/A_2_). Therefore, the smallest resolution of the measure for this system is 4.882 mm. The output precision of the system is ±1 cm, which is related to the range of the measure.

### 4.3. Error Rate

The bit error rate of the data transmission is less than 0.004%. The error of the data transmission can be checked out by the CRC (Cyclic Redundancy Check), which adds up all the data before transmission and pick up the 16 digits at the right-hand end of the row as check code to detect the data transmission. The data will be re-sent when the data goes wrong.

### 4.4. Power Consumption

The power consumption is maximal in data transmission. At least 2A transient current should be supplied because of the pulse current in emission. If the current is below 2A, the system will restart due to the insufficient voltage. The average current of the system is less than 500 mA during data transmission. The maximum energy consumption is approximate 200 mW when the water-level sensor is operated, but it is only 25 mW in power saving mode.

### 4.5. Sampling Frequency and Response Time

The sampling frequency of this system can approach 1 MHz, while response time is less than 4 ms. The embedded real-time operation system can run multiple tasks independently, which shortens the response time of the system greatly.

## 5. Discussion and Conclusions

The RWMS designed in this paper has the following advantages over other similar water-level monitoring systems available at home and abroad, shown in Table 1: (1) The software and the hardware are researched and developed independently. They can not only tremendously ensure a stable, expansible and reliable system, but also reduce the expenses. The cost is half that of the similar products available in China and one-sixth of the similar overseas products with a conservative estimation. The hardware is designed independently to ensure reasonable hardware settings and a strong anti-jamming system, especially for the unattended and changeable environments. (2) The system possess good real time performance and synchronicity. The data obtained from this system’s sensor modules are written into the memory of the base station, which are transmitted to the data center immediately realize the real-time, fast, and wireless remote acquisition of data. The system will obtain a better synchronization when the data have been transmitted through the satellite and multiple sites orderly. (3) The system has several methods to release and process the data. They can be released through the mobile mode, the WEB mode and the web page browsing. Besides, the trend analysis can be realized through the data center.

**Figure 11.**
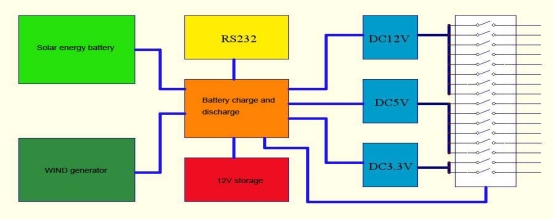
Embedded intelligent power management system.

The RWMS can be further improved on the following aspects: (1) Combination of more nodes. Users can obtain the data in large-scale by placing the nodes orderly and optimizing the structure of the sensor network. (2) Intelligent power management. The current system uses storage batteries to provide power, but this method has its limitations in inaccessible places. The self-developed embedded intelligent power management is based on a low-temperature rechargeable battery, solar energy and wind power generation, as shown in Figure 11.

This intelligent power management system can work in gear without wind and solar. It can take certain actions to protect the system, such as reducing the sampling frequency or cutting off the high energy sensors and so on. (3) Integration of the system with RS (remote sensing). RS is the macroscopic method to study Nature. Integrating the RS with the real-time data is an important direction in the future and has important theoretic value and practical significance.

## Figures and Tables

**Table 1.** Comparation of different systems.

**System**	**Cost**	**Real-time**	**Synchronicity**	**Remote Upgrade**	**Expandability**	**Customizability**	**Giving an alarm**
Water Environment Monitoring System^[6]^	L	Y	Y	N	Y	U	N
Micro-environmental monitoring system^[7]^	U	Y	U	N	U	U	U
JWR-SJ^[1]^	H	U	N	N	U	N	N
HG35-QSW^[2]^	H	N	N	N	U	N	N
DATA86^[3]^	H	Y	N	Y	U	U	U
RWMS	L	Y	Y	Y	Y	Y	Y

Notes: L-Low, H-High, Y-Yes, N-No, U-Unknown
